# On modelling airborne infection risk

**DOI:** 10.1098/rsos.231976

**Published:** 2024-07-24

**Authors:** Yannis Drossinos, Nikolaos I. Stilianakis

**Affiliations:** ^1^ Thermal Hydraulics & Multiphase Flow Laboratory, Institute of Nuclear & Radiological Sciences and Technology, Energy & Safety, National Centre for Scientific Research “Demokritos”, Agia Paraskevi 15314, Greece; ^2^ Joint Research Centre (JRC), European Commission, Ispra, VA 21027, Italy; ^3^ Department of Biometry and Epidemiology, University of Erlangen-Nuremberg, Erlangen, Germany

**Keywords:** infectious diseases, SARS-CoV-2 transmission, aerosol, infectious respiratory particle (IRP), Wells–Riley infection risk model, Gammaitoni–Nucci infection risk model

## Abstract

Airborne infection risk analysis is usually performed for enclosed spaces where susceptible individuals are exposed to infectious airborne respiratory droplets by inhalation. It is usually based on exponential, dose-response models of which a widely used variant is the Wells–Riley (WR) model. We revisit this infection-risk estimate and extend it to the population level. We use an epidemiological model where the mode of pathogen transmission, airborne or contact, is explicitly considered. We illustrate the link between epidemiological models and the WR and the Gammaitoni and Nucci models. We argue that airborne infection quanta are, up to an overall density, airborne infectious respiratory droplets modified by a parameter that depends on biological properties of the pathogen, physical properties of the droplet and behavioural properties of the individual. We calculate the time-dependent risk of being infected for two scenarios. We show how the epidemic infection risk depends on the viral latent period and the event time, the time infection occurs. Infection risk follows the dynamics of the infected population. As the latent period decreases, infection risk increases. The longer a susceptible is present in the epidemic, the higher its risk of infection for equal exposure time to the pathogen is.

## 1. Introduction

The determination of the risk of airborne infection during an epidemic is an important quantitative indicator that, among others, influences decisions of public health authorities on intervention strategies and their implementation, including vaccine administration. It contributes, also, to individual decisions on whether to accept recommendations on physical distancing, proper wearing of face masks and mobility restrictions. Estimates of the risk associated with airborne respiratory-pathogen infection have become numerous since the beginning of the coronavirus disease 2019 (COVID-19) pandemic.

Most airborne infection-risk analyses during the COVID-19 pandemic concentrated on risk calculations in small, enclosed spaces within which susceptible individuals are exposed to infectious airborne respiratory droplets by inhalation for a brief period. For example, the probability of infection owing to the severe acute respiratory syndrome coronavirus 2 (SARS-CoV-2) has been estimated in numerous private and public micro-environments [1–7]. The majority of these risk analyses were based on the exponential, dose-response Wells–Riley (WR) model or its variants.

The WR [8–10] model is a deterministic exposure model, based on the probabilistic airborne infection model proposed by Wells [11]. Wells introduced the quantum of airborne infection^1^ to be a discrete entity of the infectious dose that would, according to a Poisson distribution, give a 63.21% probability of infection [12] or, in modern terminology, the Infectious Dose ID${\;}_{63.21}$. Riley *et al*. [8], expanding on Riley [13] and using Wells’ quantum of infection, introduced the average number of quanta inhaled during an individual’s exposure to an airborne pathogen in an exponential dose-response model. They, thus, proposed a model for the risk of airborne infection in an indoor environment. They assumed that the micro-environment is homogeneous, and hence infection quanta were uniformly distributed, and that the quantum concentration and the ventilation conditions were at steady state. The resulting steady-state model is commonly referred to as the WR model. Moreover, they took I, the number of infectors, constant during exposure, but not so the number of susceptibles S, assuming that the viral latent period, the time between being infected and becoming infectious, is much longer than the exposure time, the duration individuals are exposed to the pathogen. An important generalization of the WR model was proposed by Gammaitoni and Nucci (GN) [14]. They removed the assumption of steady-state quantum concentration to generalize it to time-dependent quanta concentrations.

One of the characteristics of the WR model is that it uses input from aerosol science to estimate viral transmissibility in, e.g. calculations of the generation rate of the quanta of infection, and their removal rate via, e.g. gravitational settling or indoor-air ventilation. Human behaviour, however, is naively modelled by the lumped parameter of exposure time. The model, being an individual-level model, and in contrast to compartmental epidemiological models, does not consider the total population N. Instead, the enclosed-space volume V determines the system scale.

Infection risk estimates in larger, including closed or semi-closed, populations and at longer, but intermediate, spatial and temporal time scales than those investigated by micro-environmental models are equally important. Envisioned intermediate spatial scales are those encountered in, e.g. hospitals, prisons, ships and nursing homes. Mesoscopic epidemiological models address these scales. The Susceptible-Infected-Recovered model with explicit modelling of the dynamics of the pathogen-carrying agent (SIR-DC model [15,16]) is one such model. The model considers that the pathogen-carrying agents, the pathogen ‘vector’, are either airborne infectious respiratory droplets (denoted by D as the corresponding transmission mode is via inhalation of airborne droplets) or deposited droplets (denoted by C, since the corresponding transmission mode is direct or indirect contact). In modelling the dynamics of the pathogen agent the SIR-DC model differs from the standard SIR model where the mode of pathogen transmission is only *implicitly* considered. Moreover, similarly to SIR-like models and contrary to micro-environmental models, the SIR-DC model is a population-level model.

Macroscopic models, on the other hand, address much larger populations and much longer temporal and spatial scales, for example, country-wide and province scales [17–19] or regional scales [20]. At such scales, micro-environmental dynamics is not modelled. Instead, the intricate dynamics of respiratory droplets and other micro-environmental processes is implicitly incorporated through effective transmission rates or parameters, via a procedure akin to coarse-grained descriptions of physical systems [16].

Noakes *et al*. [10] presented an early attempt to reconcile the WR expression with a standard SIR compartmental epidemiological model. We use an extended version of the SIR-DC droplet model to revisit the derivation and to estimate what we shall refer to as the epidemic infection risk, the infection risk during an epidemic. We establish firmly the connection between compartmental epidemiological models and micro-environmental risk models, like the WR model and its Gammaitoni–Nucci generalization, and the relevance of respiratory droplet dynamics. One of the essential observations is that neither the GN nor the WR model considers time-dependent changes in the infected population. In establishing this connection, we elucidate the meaning of the term *quantum of airborne infection*, introduced by William Firth Wells in his classic 1955 book [11]. As argued in [21], this term has often been interpreted confusingly.

Lastly, we calculate the epidemic infection risk, the probability of being infected, the event, at a specific time later than an arbitrarily chosen time during the epidemic. In essence, the question we address is: what is the probability at time t to be infected at a later time t+δt. The time t, with respect to the beginning of the epidemic, is the time at which risk is evaluated, and the time t+δt is the event time. In our numerical simulations, we calculate this probability as a function of the pathogen latent period and as a function of the difference between the event time and the time the infection risk is calculated, δt, the risk time.

## 2. Infection probability in compartmental epidemiological models

The epidemic infection risk $P(t,\delta t;\langle \tau_{\text{exp}}\rangle )$, for an average daily exposure time $\langle \tau_{\text{exp}}\rangle$, is the probability at time t from the beginning of an epidemic to be infected at a future time t+δt as specified by the event, i.e. the infection. The time difference δt is the time interval that determines how the infection risk at t depends on the evolution of the epidemic at t+δt. We shall refer to it as the risk time. The average daily exposure time is an estimate of a susceptible’s daily exposure to the pathogen: it is taken to be constant during an epidemic. For example, in the SIR-DC model, the population dynamics depends on an average, daily exposure time which is embedded in the transmission rate (see Supplementary Material (SM)). In the standard SIR model, the dynamics depends on the average exposure time implicitly via, e.g. the daily number of contacts between susceptible and infected individuals. As the average exposure time is taken to be constant, and the dependence of the probability on it is implicit, we shall simplify notation and refer to the infection probability as P(t,δt) and the number of susceptibles as S(t) (instead of $S(t;\langle \tau_{\text{exp}}\rangle )$.

The epidemic infection risk expressed in terms of the number of susceptible individuals S is their relative change [10,22] in the period [t,t+δt],


(2.1) $$P\left(t,\delta t\right)=\frac{S\left(t\right)-S\left(t+\delta t\right)}{S\left(t\right)}.$$

Equation (2.1) provides the connection between epidemiological compartmental models and infection-risk models. Any epidemiological model that calculates S(t) can be used to calculate the probability of infection. In fact, the two approximations we will consider, WR and GN, refer to different approximate ways to calculate S(t+δt).

The epidemic infection risk depends explicitly on the two-time scale (t,δt) and implicitly on $\langle \tau_{\text{exp}}\rangle$, in stark contrast to WR-based risk estimates that apparently depend on a single time scale, the time interval a susceptible is exposed to the pathogen. The time t, the time at which risk is calculated, may be additionally considered in WR models as it determines the initial number of infectors. The risk time δt, i.e. the difference between event and the time risk is evaluated, is analogous to the exposure time in WR-based models, in that it determines the interval over which the epidemiological populations evolve, and thus the time after t that the number of susceptibles S(t) is to be determined. In the interval [t,t+δt], the population S(t) evolves according to model dynamics, herein taken to be the SIR-DC dynamics or the approximate dynamics the WR or GN models. In WR-like models, the exposure time is the time over which the number of susceptibles changes, and thence the time scale that determines infection risk.

Another important difference between SIR-like and WR-based models is that the overall time scale of compartmental epidemiological models is of the order of days or months, whereas the time scale of WR-based risk analyses is of the order of hours.

## 3. Susceptible-Exposed-Infected-Recovered model with transmission modes (SEIR-DC)

### 3.1. Droplet model

As other respiratory viruses, the SARS-CoV-2 virus exhibits a latent period. During the latent period $\tau_{\text{lat}}=1/\sigma$ exposed individuals are infected but not infectious. Accordingly, we generalize the SIR-DC model of infectious disease transmission via infectious respiratory droplets [15] by adding an exposed population compartment E. The SIR-DC model is a population epidemiological model where individuals can move between the three standard epidemiological compartments of the SIR model. In addition, it models the dynamics of the pathogen-carrying agent. Infection does not occur via direct I↔S interaction: this interaction, instead, is mediated by the infectious droplets, be they airborne or settled.

A note is in order on the naming of the model. It was initially referred to as SDIR [16], a name that mixes epidemiological populations (S,I,R) with the pathogen-carrying agents (D,C) that determine the transmission mode. Stimulated by [23] and their SEIR-C model, we opted to separate epidemiological populations from transmission modes. Infectious respiratory particles (IRPs, see §5) that are responsible for non-contact airborne transmission [24] are denoted by D (for droplets), whereas settled droplets that are responsible for contact transmission, direct or indirect, are denoted by C (for contact).

Bazant and Bush [25] also included the exposed population to connect the SEIR to the WR model. They, as we do herein, also considered cases of short and long latent periods of the pathogen. Our approaches differ, however, in how the epidemiological population compartments are coupled to the infectious respiratory droplets. The SEIR-DC model is defined by the following set of coupled ordinary differential equations (ODEs)


(3.1a) $$\frac{dS}{dt}=-\sum_{i=1}^{i=i_{\max}}\left(\frac{\beta_{i}^{d}}{N}D_{i}S+\frac{\beta_{i}^{c}}{N}C_{i}S\right),$$

(3.1b) $$\frac{dE}{dt}=-\frac{dS}{dt}-\sigma E,$$

(3.1c) $$\frac{dI}{dt}=\sigma E-\mu_{I}I,$$

(3.1d) $$\frac{dD_{i}}{dt}=\kappa_{i}^{d}I-\alpha_{i}^{d}D_{i},\;\;\;\;\text{for}\;\;\;\;i=1,2\ldots i_{\max},$$

(3.1e) $$\frac{dC_{i}}{dt}=\kappa_{i}^{c}D_{i}-\alpha_{i}^{c}C_{i},\;\;\;\;\text{for}\;\;\;\;i=1,2\ldots i_{\max}.$$

**Figure 1.**
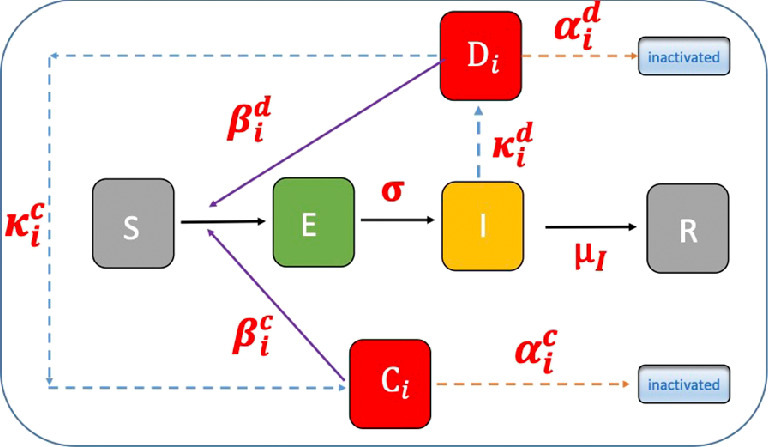
Schematic diagram of the Susceptible-Exposed-Infected-Recovered model with transmission modes, SEIR-DC (based on a figure of [16]). Infectious droplet compartments (equivalently, infectious-respiratory-particle, IRP, compartments, see §5) are denoted by $D_{i}$, airborne droplets, and $C_{i}$, settled droplets. Superscripts (d,c) denote (airborne, settled) droplets, the subscript i refers to droplets with post-evaporation diameter $d_{i}^{\text{post}}$. Infection transmission rates are denoted by $\beta_{i}^{d,c}$, droplet generation rates by $\kappa_{i}^{d,c}$, and removal rates by $\alpha_{i}^{d,c}$. The latent period is $\tau_{\text{lat}}=1/\sigma$ and the infection recovery rate $\mu_{I}$.

We do not show the equation for the recovered compartment R since the total population S+I+R=N is constant representing a closed population. A schematic diagram of the model is shown in figure 1.

The number of infectious airborne droplets of post-evaporation diameter $d_{i}^{\text{post}}$ is denoted by $D_{i}$ (number), and that of settled droplets by $C_{i}$ (number), cf. SM for a discussion of droplet evaporation and associated droplet diameters. The number of droplet classes is $i_{\text{max}}$. The rate of transition from the exposed compartment E to the infected compartment I is denoted by σ. The infection recovery rate I→R is $\mu_{I}$. Superscripts denote airborne droplet (d) and settled (c), and the subscript i denotes the droplet class specified by the post-evaporation diameter $d_{i}^{\text{post}}$. The transmission rate per infectious, airborne respiratory droplet that has been inhaled and deposited in the respiratory tract of a susceptible is denoted by $\beta_{i}^{d}$ (inverse time), whereas that of an infectious settled droplet transferred to facial membranes is denoted by $\beta_{i}^{c}$ (inverse time).

In this version of the SEIR-DC model infection by airborne respiratory droplets can solely occur via their inhalation. We do not consider infection via direct, ballistic-like deposition of expelled droplets at close proximity of the infectious individual to a susceptible. Thus, we neglect short-range conversational immediate exposure scenarios. In terms of the recently introduced terminology for the modes of transmission by the World Health Organization (WHO) [26,27], we incorporate airborne (or inhalation) transmission, but we neglect direct-deposition transmission. The original WR and GN models also only included inhalation transmission. We consider direct-deposition transmission relatively infrequent on the population level, especially if non-pharmaceutical intervention measures have been enforced. Nevertheless, direct-deposition transmission may be easily included by adding an appropriately calculated, probably via averaging computational fluid dynamics simulations, transmission rate.

The airborne droplet generation rate per infected individual (by normal oro-nasal activities—e.g. speaking, laughing, breathing—or by violent expiratory events—sneezing, coughing) is $\kappa_{i}^{d}$ (number/time) and the corresponding airborne droplet removal rate is $\alpha_{i}^{d}$ (number/time), the later including droplet removal by ventilation (if present). Settled droplets may be generated either via direct generation by an infected individual and deposition on surfaces or via deposition of airborne droplets. Direct surface deposition would introduce an additional generation term in equation (3.1e) proportional to the number of infected individuals, similar to the generation term in the airborne-droplet equation, equation (3.1d). In this version of the model, we neglect this mechanism. Instead, settled droplets are generated via deposition of airborne droplets, and specifically solely by gravitational settling, which we take to occur in still air. Hence the generation rate $\kappa_{i}^{c}=\theta_{i}(d_{i}^{\text{post}})$ (number/time) with θ the gravitational settling rate in still air. The corresponding settled droplet removal rate is $\alpha_{i}^{c}$ (number/time).

We present expressions for the transmission $\beta_{i}^{c,d}$ and removal $\alpha_{i}^{c,d}$ rates, along with justifications for our choices, in SM. We remark that the transmission and removal rates are *derived* quantities. In addition, both transmission rates depend (linearly as we argue in SM) on the average exposure time $\langle \tau_{\text{exp}}\rangle$. The SIR-DC basic reproduction number is [15,16]


(3.2) $$R_{0}^{\text{SIR-DC}}=\sum_{i=1}^{i=i_{\max}}\left(\frac{\beta_{i}^{d}\kappa_{i}^{d}}{\alpha_{i}^{d}\mu_{I}}+\frac{\beta_{i}^{c}\kappa_{i}^{c}}{\alpha_{i}^{c}\mu_{I}}\right).$$

Equation (3.2) also gives the SEIR-DC basic reproduction number, as may be shown using the arguments of [28].

### 3.2. Infectious quanta: Gammaitoni–Nucci approximation

We limit the infectious droplet classes to a single airborne droplet class $D_{1}$ (SEIR-D) as the original GN model considered only one droplet diameter. It can be shown, for example by integrating the infected population equation (3.1c), that if σδt≪1, latent period much greater than the risk time (the time the epidemiological populations evolve to calculate the infection probability), and $\mu_{I}\delta t\ll 1$, infectiousness period much greater than the risk time δt, then $dI/dt{|}_{t=t_{0}}=0$. Hence, under these limits, the number of infected at time t, here taken to be the initial time $t_{0}$, may be considered to be constant and denoted as $I_{0}$. If, in addition, we disregard the equation for the exposed population equation (3.1b), which is irrelevant over the risk time δt for the time-development of the infection, σδt<<1 (the number of E increases, but not that of I), the SEIR-D model reduces to


(3.3a) $$\frac{dS}{dt}=-\frac{\beta_{1}^{d}}{N}D_{1}S,$$

(3.3b) $$\frac{dD_{1}}{dt}=\kappa_{1}^{d}I_{0}-\alpha_{1}^{d}D_{1}.$$

The system of equations (3.3) can be compared to the GN equations (3.4) for the rate of change of the number of susceptibles and total number of quanta of infection Q in an enclosed space. The GN equations expressed in our notation read


(3.4a) $$\frac{dS}{dt}=-\frac{B}{V}QS,$$

(3.4b) $$\frac{dQ}{dt}=qI_{0}-\lambda_{\text{air}}Q,$$

where q is the quantum generation rate per infectious individual (quanta/sec), see also [10], B is the breathing rate (m${\;}^{3}$/s), and V is the space volume (m${\;}^{3}$). The parameter $\lambda_{\text{air}}$ is the quantum removal rate which in the initial formulation of the model was taken to be the ventilation rate in air exchanges per hour [14]. Since then, it has been expanded to include the rate of pathogen inactivation, droplet deposition on surfaces, inactivation owing to UV irradiation, filter penetration, mask efficiency, etc. (see also the droplet removal rates $\alpha_{1}^{d}$ used in this work and summarized in SM).

The analytical solution of equation (3.4b) is


(3.5) $$Q\left(t,\delta t\right)=\frac{qI_{0}}{\lambda_{\text{air}}}+\left(Q_{0}-\frac{qI_{0}}{\lambda_{\text{air}}}\right)exp\left(-\lambda_{\text{air}}\delta t\right),$$

where $Q_{0}$ is the initial (at time $t=t_{0}$) total concentration of the infection quanta in the enclosed space.

Even though the two sets of equations (3.3) and (3.4) are formally equivalent, their interpretation and the time scales chosen to determine infection risk differ. In our numerical simulations, we use typical time scales associated with compartmental epidemiological models, of the order of months. WR-based models in micro-environments, instead, use considerably shortened time scales, of the order of hours.

In §4, we use the GN equations to approximate the dynamics of the number of susceptibles S(t), and subsequently the infection risk according to equation (2.1). As the droplet equation (3.3b), and subsequently the susceptible equation (3.3a), may be solved analytically, in our numerical simulations we used the analytical solutions.

#### 3.2.1. What are infection quanta?

The comparison of equations (3.3) and (3.4) provides insights into the differences and formal similarities of the SEIR-D and GN models. Let the number of quanta of infection be proportional to the number of infectious respiratory droplets


(3.6) $$Q=\xi D_{1},$$

and the transmission rate proportional to the breathing rate, $\beta_{1}^{d}=B{\tilde{\beta }}_{1}^{d}$, as argued in SM. Moreover, for the purposes of this comparison, consider indoor-air ventilation as the only droplet or in the case of the GN model quantum removal process, $a_{1}^{d}=\lambda_{\text{air}}$. Their substitution into equations (3.3) and a mapping of the resulting equations to equations (3.4) determine the conversion factor ξ to be


(3.7) $$\xi =\frac{\beta_{1}^{d}}{B}\;\frac{V}{N}\equiv {\overset{˜}{\beta }}_{1}^{d}\rho_{\text{scale}},$$

where the last equation defines the scaling density $\rho_{\text{scale}}=V/N$. Hence, in this model, airborne infection quanta, up to an overall scaling density, are airborne infectious respiratory droplets modified by ${\tilde{\beta }}_{1}^{d}$, a parameter that includes the probability of infection of a lung-deposited pathogen, number of pathogens in a droplet, lung-deposition probability, and average exposure time, cf. SM. The combination of these factors converts infectious airborne droplets to infection quanta. Their generation q is similarly related to the respiratory droplet generation rate via $q=\kappa_{1}^{d}\xi$.

The mapping of the two models also manifests the different inherent system scales: the extensive variable, namely, the variable that scales linearly with the size of the system, is the volume of the enclosed space in the GN model, whereas it becomes the total population N in the SEIR-D model. The scaling density $\rho_{\text{scale}}$ implements the transition from a microscopic model, which depends on the enclosed-space volume V, to a mesoscopic epidemiological model, which depends on the total population N. This scaling is reminiscent of the scaling proposed in [17] to transition from an ODE to a partial differential equation (PDE) epidemiological model.

Care should be exercised in interpreting $\rho_{\text{scale}}$: if V is taken to refer to a mesoscopic volume, then the GN model is essentially extended to much greater scales. If N is taken to be the number of individuals in an enclosed, micro-environment the SIR-D model is restricted to smaller scales; however, in that case, it may not be considered a proper compartmental epidemiological model. These considerations have important repercussions on the choice of model parameters and risk times in micro- or mesoscale models.

### 3.3. Wells–Riley approximation

Reference [29] considered analytically the very common limit where the duration of infectiousness of an infected individual $T_{I}=1/\mu_{I}$ is significantly longer than the lifespan of the airborne pathogen $T_{p}=1/\alpha_{1}^{d}$, i.e. when $\rho_{1}\equiv \mu_{I}/\alpha_{1}^{d}=T_{p}/T_{I}\ll 1$. For appropriately chosen non-dimensional variables, denoted by tilde, [29] the quasi-steady-state limit is defined as $\rho_{1}d{\tilde{D}}_{1}/d\tilde{t}=0$, which implies ${\overset{˜}{D}}_{1,\text{qss}}={\overset{˜}{I}}_{\text{qss}}$, or in terms of the original variables $D_{1,\text{qss}}=\left(\kappa_{1}^{d}/\alpha_{1}^{d}\right)I_{\text{qss}}$. Note that the quasi-steady-state condition does not imply that the number of infected individuals is constant, $dI/dt{|}_{\text{qss}}\neq 0$, that is $I_{\text{qss}}$ is *time dependent*.

The substitution of the steady-state ($I,D_{1}$) relationship in the original equations (3.1) gives the quasi-steady-state limit of SEIR-D,


(3.8a) $$\frac{dS_{\text{qss}}}{dt}=-\frac{\beta_{1}^{d}\kappa_{1}^{d}}{\alpha_{1}^{d}N}I_{\text{qss}}S_{\text{qss}},$$

(3.8b) $$\frac{dE_{\text{qss}}}{dt}=\frac{\beta_{1}^{d}\kappa_{1}^{d}}{\alpha_{1}^{d}N}I_{\text{qss}}S_{\text{qss}}-\sigma E_{\text{qss}},$$

(3.8c) $$\frac{dI_{\text{qss}}}{dt}=\sigma E_{\text{qss}}-\mu_{I}I_{\text{qss}}.$$

In the quasi-steady-state limit, the dependence on the number of infectious droplets $D_{1}(t)$ disappears.

As before, in the previously considered double limit, $\sigma \delta t,\mu_{I}\delta t\ll 1$, we can neglect the equation for the exposed population, equation (3.8b), and take the number of infected individuals constant, $I_{\text{qss}}=I_{0}$. The analytical solution of the resulting model leads directly to the WR approximation of the SEIR-D model


(3.9) $$P_{\text{WR}}^{\text{SEIR-D}}\left(t,\delta t\right)=1-exp\left(-\frac{\beta_{1}^{d}\kappa_{1}^{d}}{\alpha_{1}^{d}N}I_{0}\delta t\right).$$

Hence, the WR equation is obtained from the quasi-steady-state SEIR-D equations in the triple limit of latent period and infectiousness period longer than the time scale of observation and $\rho_{1}\ll 1$. Substitution of $k_{1}^{d}=q/\xi$, along with equation (3.7), and $\alpha_{1}^{d}=\lambda_{\text{air}}$ in equation (3.9) leads to the WR infection probability as usually written.

Of course, the WR approximation to the GN model may be easily obtained by setting dQ/dt=0 in equation (3.4b). The steady-state quantum concentration, then, becomes $Q_{ss}=qI_{0}/(\lambda_{\text{air}})$, leading via equation (3.4a) to the number of susceptibles and, thus, to the WR infection risk. However, the alternative derivation for the WR approximation we presented in terms of the quasi-steady-state solution of the SEIR-D model specifies the region of validity of the approximation, instead of arbitrarily setting dQ/dt=0.

## 4. Numerical results

We performed numerical simulations of the SEIR-D model, equations (3.1), to investigate the effect of the event time t+δt, via δt, and of the latent period $\tau_{\text{lat}}$ on epidemic infection risk. As mentioned, we address the question of what is the probability at time t, any arbitrarily chosen time during the epidemic, to be infected at a future event time t+δt, given the evolution of the pandemic till t+δt. We also investigate numerically and analytically the validity of the GN, equations (3.3), and WR, equation (3.9), approximations to the SEIR-D population dynamics.

For the simulations, we used parameters related to the COVID-19 pandemic, e.g. individual behaviour characteristics in addition to physico-chemical and biological properties of the SARS-CoV-2 virus. We note, though, that we do not attempt to reproduce a COVID-19 scenario as in our attempt to present the minimal model that reduces to the GN or WR models we do not consider the asymptomatic stage of the disease.

We used two airborne infectious droplet classes of post-evaporation diameter $d_{i}^{\text{post}}=2.05,82.13$μm (i=1,2). As generally accepted for SARS-CoV-2 [30], the pathogen concentration was taken to be droplet-size dependent. We opted to limit the airborne infectious-respiratory droplet classes to two and not to simulate settled droplets to render easier the interpretation of our results: either condition can be easily relaxed. The evaporation factor [16], $d_{i}^{\text{post}}=z_{\text{evap}}d_{i}^{\text{pre}}$, was set to $z_{\text{evap}}=0.40$. Airborne droplet generation rates were taken to correspond to speaking. A complete list of model parameters is presented in SM.

Individual behaviour determines a number of model parameters. We considered the contact rate, the number of susceptible-infected individual encounters, to be c=18 per day [31]. The exposure time of a susceptible with an infectious droplet, i.e. the breathing time during a S↔I encounter, was taken to depend on the droplet size: $\tau_{d_{1}}=25$ min and $\tau_{d_{2}}=1$ min. Thus, the average exposure time per day of a single susceptible is $c\times (\tau_{d_{1}}+\tau_{d_{2}})=7.8$ h per day.

**Figure 2.**
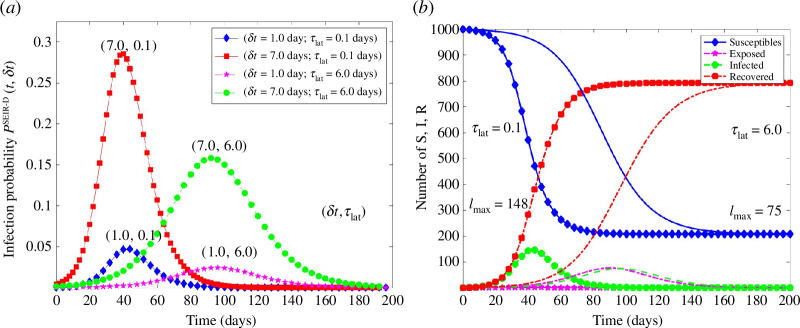
(*a*) Epidemic infection probability, i.e. the probability at t to be infected at a future time t+δt, according to the SEIR-D model. Curves were calculated for two risk times (δt=1,7 days) and two latent periods ($\tau_{\text{lat}}=0.1,6$ days). Two airborne-droplet classes were considered, ($d_{i}^{\text{post}}=2.05,82.13\;\mu$m (i=1,2)), susceptible-infectious droplet encounters per day were taken to be c=18, exposure time for each $S↔D_{i}$ (i=1,2), encounter $\tau_{d_{1}}=25$ min and $\tau_{d_{2}}=1$ min, leading to a total daily susceptible-infectious droplet average exposure time of $\langle \tau_{\text{exp}}\rangle =7.8$ h (per day). The ventilation rate was taken to be $\lambda_{\text{air}}=0.2$ air exchanges per hour [1]. Total population N=1000. (*b*) Corresponding dynamics of the two epidemics. The left curves (filled symbols) correspond to the short latent period, $\tau_{\text{lat}}=0.10$ days, with I peaking at t≈43 days, and no discernible exposed population. The right curves (lines, no symbols) show the epidemic for the long latent period $\tau_{\text{lat}}=6$ days, with I peaking at t≈96 days, and an appreciable exposed population.

Figure 2 summarizes the main results of four simulations to determine the probability at time t that infection occurs at the event time t+δt. We used two latent periods $\tau_{\text{lat}}=0.1$ days (short) and $\tau_{\text{lat}}=6.0$ days (long), along with a short δt=1.0 day and a long relative risk time interval δt=7 days. The left panel shows the calculated infection probabilities for each scenario. Two groups of curves may be identified: for the short latent period, the infection probability peaks at about $t_{\text{peak}}\approx 43$ days, whereas for the long latent period, the peak occurs at $t_{\text{peak}}\approx 96$. Within each group of curves, infection risk increases with increasing risk time.

The qualitative behaviour of infection risk may be understood by considering the dynamics of the epidemic, described by the time-dependent number of S,E,I and R shown in the right panel of figure 2. The four curves on the left (filled symbols) correspond to the short latent period, whereas those on the right (no symbols) to the long latent period. We also present the maximum number of infected individuals $I_{\text{max}}$ for each epidemic. For the short-latent period epidemic, the number of exposed individuals is very small, not discernible on the figure, whereas for the long-latent period epidemic, the number of exposed individuals is comparable to the number of infected. In fact, before the I maximum, E>I, whereas afterwards I>E. Even though not clearly discernible, the number of exposed individuals E peaks earlier than the number of infected I.

We find that infection risk follows the time-dependent behaviour, the dynamics, of the infected individuals I. As the number of infected increases, infection risk increases, and vice versa. As the latent period decreases infection risk increases, since the number of infected increases. In addition, for the same pathogen (latent period constant) infection risk increases with increasing risk time δt, i.e. with increasing difference between event time and time t, the time risk is evaluated. In fact, this is a general result: the longer the epidemic evolves and the longer a susceptible is present in it, the more likely the individual is to be infected.

**Figure 3.**
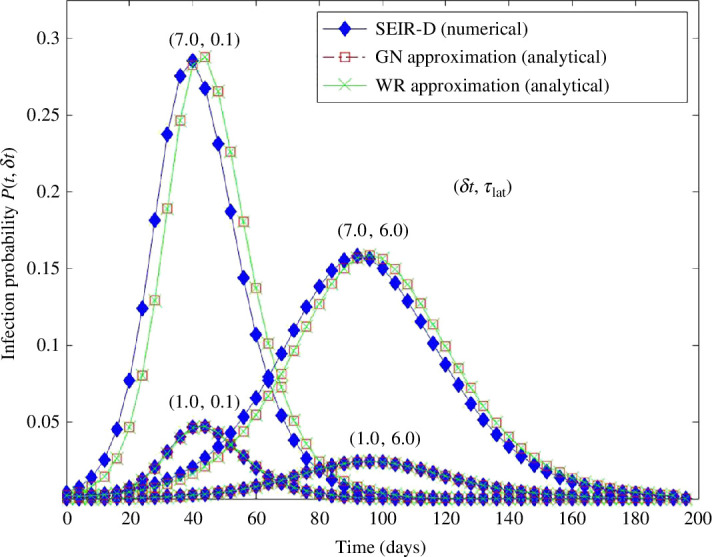
Epidemic infection probability calculated via the SEIR-D model and its GN, via appropriately modified equations (3.4), and WR approximations, via equation (3.9). The four ordered pairs associated with each graph triplet are specified by ($\delta t,\tau_{\text{lat}})$. For all the simulations the GN and WR limits were identical: they differed from the SEIR-D model predictions only for the long risk time (δt=7 days). See the main text for an explanation.

The validity of the GN and WR approximations to the SEIR-D dynamics is investigated numerically in figure 3. Four groups of curves are shown, each corresponding to the ordered pair ($\delta t,\tau_{\text{lat}})$. For each pair choice, we plot the SEIR-D infection risk calculated via the numerical solutions of equations (3.1) (filled blue diamonds), infection risk calculated via the GN approximation and described in §3.2 (square, unfilled symbols), and via the WR approximation $P_{\text{WR}}^{\text{SEIR-D}}$ as calculated via equation (3.9) (cross, continuous line).

Two observations are in order. For the epidemics considered, the GN and WR limits are identical. Whether the two limits would differ depends on the airborne droplet removal rate $\alpha_{1}^{d}$ (and hence on the dimensionless parameter $\rho_{1}=\mu_{I}/\alpha_{1}^{d}$).^2^ The importance of the removal rate is apparent from the analytical solution of the droplet equation equation (3.3b). Its time-dependent part, which is formally identical to the second term of equation (3.5), determines the difference between the steady-state and non-steady-state model. It vanishes as $\alpha_{1}^{d}\delta t\gg 1$, a condition satisfied for all cases considered. The same observation holds for the GN-WR comparison. If ventilation is the dominant aerosol removal process, for $\lambda_{\text{air}}\delta t\gg 1$ the two models become identical. Hence, for high ventilation rates, the difference between the steady and non-steady-state quantum concentration models decreases or even vanishes.

The other observation is that for the short risk time δt=1 day all three calculations predict the same infection risk, irrespective of the viral latent period. The calculated risks differ for the long risk time, the difference increasing with the latent period decreasing, i.e. as $\tau_{\text{lat}}\to 0$. For short latent periods, the time dependence of the infected I(t) can not be neglected. As the number of I increases, i.e. before the maximum of the number of infected, the GN and WR approximate dynamics underestimates infection risk, whereas the opposite holds when dI/dt<0. This arises because when the number of I increases more infectious droplets are generated than predicted for a constant $I_{0}$ leading to a larger infection probability, and vice versa.

In an attempt to investigate the GN-WR difference, we considered an extreme case of the SEIR-D model by shortening the model time scales from days to hours. The calculated infection risk, not shown, behaved as described in the previous paragraphs, confirming the initial observation that even for short risk times, e.g. δt=12 hours, the condition $\alpha_{1}^{d}\delta t\gg 1$ remained valid. We note that the GN and WR models may, however, differ in micro-environmental simulations if the necessary condition, e.g. $\alpha_{1}^{d}\delta t\ll 1$ is satisfied.

## 5. Discussion

We presented a model, SEIR-DC, to calculate epidemic infection risk, the probability that at an arbitrarily chosen time t during the epidemic infection occurs at a future event time t+δt. The SEIR-DC model consists of the standard epidemiological populations of the Susceptible-Exposed-Infected-Recovered (SEIR) populations coupled to the dynamics of the pathogen-carrying agent, and hence to the pathogen transmission mode. The pathogen-carrying agent is taken to be either airborne infectious respiratory droplets (D), as in the case of airborne infections such as COVID-19 or influenza, or settled droplets responsible for (direct or indirect) contact transmission (C). The model provides a connection between SEIR-like epidemiological models and infection-risk models based on Wells-Riley (WR) [8] models, including the non-steady state generalization proposed by Gammaitoni and Nucci (GN) [14]. In fact, the SEIR-DC model may be viewed as a generalization of the GN model to the population level for arbitrary viral latent periods. We emphasized the importance of system scales, since both the GN and WR models are individual-level models that describe infection risk in enclosed micro-environments. SEIR-like models instead are population-level models.

A justification of our choice to describe the pathogen-carrying agents as *infectious respiratory droplets* is required. The mode of transmission of respiratory viruses has been debated heatedly since the beginning of the COVID-19 pandemic. Part of the confusion stems from the use of the words aerosol and droplet which have different meanings in the biomedical and aerosol-science communities. For example, in the biomedical community, it was (pre-COVID-19) common to refer to aerosols as small respiratory droplets, usually smaller than 5 microns, responsible for respiratory-virus transmission via inhalation, and to droplets as larger droplets, of particle diameter larger than 5 microns, responsible for transmission via ballistic deposition on facial mucous membranes. In the aerosol science community, aerosol is a suspension of solid or liquid particles in a gas, the suspension remaining stable long enough to be measured. A droplet is a liquid particle suspended in a gas [32].

Fortunately, a consensus, enshrined by the recent World Health Organization (WHO) technical report [26] ‘Global technical consultation report on proposed terminology for pathogens that transmit through air’ on a uniform (across disciplines) terminology is slowly emerging.^3^ The report proposes that the agents that carry the infectious pathogens in expelled, primarily water-containing, particles of a wide range of sizes be referred to as *infectious respiratory particles* (IRPs), thereby eliminating the artificial dichotomy between aerosols and droplets. We think this is an apt term and a useful compromise that will probably help improve understanding between different communities and alleviate remaining differences.^4^ In fact, we use the term particle occasionally in this work. Nevertheless, we decided to retain the term droplet as it has a precise definition in aerosol physics. For extensive critical reviews of the terminology used to categorize respiratory virus transmission see, for example, [16,34,35]. In addition, [24] made the early suggestion that the diameters of IRPs, and their associated infectivity, should be considered a continuum without artificial particle-size demarcations, anticipating the recent WHO reports [26,27].

We argued that for long latent periods of the pathogen, the SEIR-DC model reduces to a set of equations that are reminiscent of the GN equations. Their comparison identified airborne infection quanta as airborne infectious respiratory droplets (equivalently, infectious respiratory particles - IRPs) modified by a scaling density and, more importantly, by a combination of parameters that include biological properties of the pathogen (size-dependent pathogen droplet concentration, probability of infection owing to a deposited infectious droplet), physical properties (lung-deposition probability) and behavioural properties (exposure time). We noted that the SEIR-DC epidemiological model depends on the total population N, whereas both the WR and GN models consider much smaller scales in terms of the enclosed volume V. We identified the scaling density as the factor to transition from one class of models to the other, and we discussed how this density allows a generalization of micro-environmental models.

We performed numerical simulations of two scenarios for an epidemic specified by a short and a long latent period and driven by two classes of airborne infectious droplets. Model parameters were based on properties of the SARS-CoV-2 virus, even though we do not claim to model specifically the SARS-CoV-2 transmission dynamics with all its specificities. However, we note that the SARS-CoV-2 transmission dynamics reflects those of a range of airborne infections such as influenza.

Our numerical simulations determine the risk at any time t to be infected at a future event time t+δt, as determined from the evolution of the epidemic in the time interval [t,t+δt]. We were particularly interested in the dependence of the calculated epidemic infection probability on the event time t+δt via δt. We found that infection risk follows the dynamics of the infected population: as the number of infected increases so does the risk, and vice versa. As the latent period decreases, infection risk increases. Moreover, as the difference between the event and the time at which risk is evaluated increases, i.e. as the risk time increases, infection risk increases. Equivalently, the longer the epidemic evolves, the longer an individual is present in an epidemic, the more likely that the individual gets infected, and hence infection risk increases.

The WR and GN approximations of the SEIR-D dynamics reproduced accurately calculated infection risk. Differences arose for large time intervals δt (δt=7 days), increasing with decreasing viral latent period. We remarked that the WR and GN approximate forms of the epidemic infection risk were almost identical for all our simulations. This arises when the droplet removal rate $\alpha_{1}^{d}$ is much greater than the inverse risk time, i.e. $\alpha_{1}^{d}\delta t\gg 1$. In fact, this is a general result suggesting that with increasing droplet removal rates, for example via increased ventilation rate, the WR-calculated airborne infection risk with a steady-state quantum concentration provides an excellent approximation to the GN-calculated infection risk with non-steady-state quantum concentrations.

The WHO recently adapted the classification of pathogens transmitting through the air, such as SARS-CoV-2, to conclude that they are predominantly airborne, in the sense that they may be inhaled as they remain airborne for hours maintaining their infectious potential. This decision is part of a broader discussion about the importance of airborne transmission as an etiological mode of respiratory-pathogen transmission and which pathogens are responsible for it. The WHO, by introducing the terminology infectious respiratory particles (IRPs), acknowledges that the distinction between droplets and aerosols is arbitrary, an observation that has been repeatedly, and over the years, pointed out by the scientific community studying influenza, see, for example, [24].

Concurrently to eliminating this artificial dichotomy, the WHO does not endorse the terminology airborne transmission for transmission of pathogens spreading through air. Instead, it introduces *through the air* transmission as an all-encompassing transmission mode for ‘pathogens travelling through or being suspended in the air’ [27]. Through-the-air transmission includes airborne (or inhalation) transmission via inhalation of IRPs and direct deposition whereby IRPs travel a short distance (through the air) before contacting the mouth, nose, or eyes of an individual [26,27]. We suspect that this distinction between airborne and direct-deposition transmission may contribute more to confusion than clarity, possibly feeding another continuous debate around the term airborne. Given the need for terms that are scientifically sound and help public health decision-makers to act properly, the term *airborne* would have been more appropriate to fulfilling both needs. Irrespective, the new proposed classification of respiratory-virus transmission modes provides a welcome and considerable improvement and advancement over previous classifications.

The SEIR-DC model, as an SIR-based level model, is a population-level model specified by a coupled set of ODEs. As such its validity and applicability are limited to a range of spatial and temporal scales. During the COVID-19 pandemic, infection risk in indoor environments was extensively investigated. Diverse modeling approaches can be used to capture pathogen transmission dynamics in indoor environments such as boarding schools, hospitals, nursing homes, military camps, prisons and cruise ships. The population in these settings is (semi-) closed, and pathogen transmission may be addressed by deterministic epidemic models that describe the temporal development of the epidemics via the interaction of susceptible and infected individuals. Deterministic approaches can be refined by explicitly incorporating expiratory droplets [15], or even expanded to investigate airborne transmission in spatially homogeneously or heterogeneously distributed susceptibles [36]. However, these models cannot be used in very small indoor environments, such as a unit of the above-mentioned settings (e.g. room in a hospital or a nursing home) and for relatively short (with respect to the duration of an epidemic) temporal scales (hours versus days). For such scales, different modelling approaches should be used. For example, computational fluid dynamics simulations provide a detailed description of the air flow and the concomitant particle transport, thereby allowing detailed descriptions of the motion of IRPs, and thus of pathogen transmission in very small spaces. Alternatively, models based on stochastic implementations of infectious-susceptible interactions, like agent-based models, may be used. The WR model and its variant belong to the category of indoor-environment modelling. The choice of the model depends on the spatial and temporal scales under investigation.

The comparative analysis presented here bridges the gap and provides the missing links in the mathematical relationship between individual infection risk models and associated population-based models. The corresponding insights allow for a more nuanced epidemiological interpretation of infectious disease outbreaks.

## Data Availability

This article has no additional data. Supplementary material is available online [37].

## Footnotes

^1^
The choice of the word quantum refers to the discrete nature of the carriers of the airborne infection, i.e. the infectious respiratory droplets (or particles, see the Discussion section). Wells might have been paying homage to the great successes of quantum mechanics, universally accepted by the time he introduced the notion of a quantum of infection.

^2^
We restrict the analysis to a single airborne droplet class, for simplicity: the arguments are easily generalized.

^3^
See, also, the more technical description of the associated WHO-sponsored multi-box model to quantify the risk of SARS-CoV-2 airborne transmission [27] and its online implementation [33]. The model incorporates recently acquired knowledge on respiratory virus transmission and non-pharmaceutical interventions (e.g. masks and respirators) to extend the Wells-Riley model.

^4^
An additional argument for the term particle over droplet is that under conditions of low relative humidity, for example indoors during winter, a droplet may loose most, but not all, its water via evaporation, with efforescence (crystallization) occurring within the droplet, leading to a drier particle.
